# SLEEVE GASTRECTOMY IN PATIENTS WITH MORBID OBESITY AND HIV

**DOI:** 10.1590/0102-6720201600S10030

**Published:** 2016

**Authors:** José Máximo Costa PINTO, Marianna Gomes Cavalcanti Leite de LIMA, Ana Luiza Melo Cavalcanti de ALMEIDA, Marcelo Gonçalves SOUSA

**Affiliations:** Department of Surgery, Paraíba Federal University, João Pessoa, PB, Brazil.

**Keywords:** Gastrectomy, Morbid obesity, HIV

## Abstract

**Introduction:**

It is estimated that there are nearly 40 million people with the human immunodeficiency virus (HIV) worldwide. Due to the advent of antiretroviral drugs, it has been observed increasing in obesity and metabolic rates among patients undergoing treatment. Thus, numerous surgical therapies for weight loss are proposed for continuous improvement in health of patients with HIV, being the vertical gastrectomy an option for intact intestinal transit.

**Objective:**

To evaluate the applicability of the vertical gastrectomy in patients with morbid obesity and HIV.

**Methods:**

Was conducted a systematic review of the literature, in the electronic databases Scopus, Pubmed, Cinahl, Scielo, Cochrane and Lilacs, from 1998 to 2015. MeSH headings used in data collection were "Gastrectomy" and "Morbid obesity" being combined with the descriptor "HIV". Were found 2148 articles in Scopus, 1234 in PubMed and 784 in Cinahl. The articles were analyzed by the Jadad Quality Scale, being reduced to 40 articles, subsequently reassessed using an elaborated form by the Critical Appraisal Skills Programme (CASP), reaching 12 articles in the end.

**Conclusion:**

It was found that vertical gastrectomy constitutes a safe and effective method, with low mortality and low rate of postoperative complications, being recommended as surgical technique in patients with obesity, HIV and comorbidities.

## RESUMO

**Introdução:**

Estima-se que haja quase 40 milhões de pessoas com o vírus da imunodeficiência adquirida (HIV) no mundo. Com o advento dos antirretrovirais, observou-se aumento da obesidade e de taxas metabólicas nos pacientes em tratamento. Assim, inúmeras terapias cirúrgicas para a perda de peso estão sendo estudadas para a melhoria contínua da saúde dos pacientes com HIV, sendo a gastrectomia vertical uma opção de trânsito íntegro.

**Objetivo:**

Avaliar a aplicabilidade da gastrectomia vertical em pacientes com obesidade mórbida e HIV.

**Métodos:**

Foi realizada revisão sistemática de literatura, de artigos publicados nas bases eletrônicas de dados Scopus, Pubmed, Cinahl, Scielo, Cochrane e Lilacs, no período de 1998 a 2015. Os descritores do MeSH utilizados na coleta dos dados foram "Gastrectomia" e "Obesidade mórbida", sendo combinados com o descritor "HIV".

**Resultados:**

Foram encontrados 2.148 artigos na Scopus, 1.234 no Pubmed e 784 na Cinahl. Os artigos foram analisados pela Escala de Qualidade de Jadad, reduzindo para um total de 40 artigos, os quais foram posteriormente reavaliados, utilizando um formulário elaborado pelo Critical Appraisal Skills Programme (CASP), chegando ao montante de 12 artigos ao final.

**Conclusão:**

Verificou-se que a gastrectomia vertical constitui método seguro e eficaz, apresentando baixa morbimortalidade e baixo índice de complicações pós-operatórias, sendo técnica cirúrgica segura para uso em pacientes com obesidade, HIV e comorbidades.

## INTRODUCTION

The acronym AIDS (Acquired Immunodeficiency Syndrome) is used to identify the disease caused by HIV (Human Immunodeficiency Virus). It is classified as a sexually transmitted infection and the diagnosis is favored by a decrease in CD4+ T lymphocyte levels below 200 cells/mm^3^ of blood and the onset of opportunistic infections^6^ ^,^ ^7^.

The United Nations Programme on HIV and AIDS estimated, in 2014, 36.9 million people living with the virus worldwide. This index remains high, even after a 35% reduction in new infections since 2000^17^.

For decades, HIV has been intrinsically linked to death. However, with the advent of Highly Active Antiretroviral Therapy, or HAART, it became possible to prevent malnutrition, weight loss and other opportunistic diseases. In contrast, has contributed to obesity, abdominal fat accumulation and metabolic changes, associated to better conditions on quality of life^13^.

The World Health Organization (WHO) defines morbid obesity as the excessive fat in people who have body mass index (BMI) greater than 40 kg/m^2^, which is becoming common in patients with HIV. Faced with this problem, numerous surgical therapies for weight loss are being used for the continuous improvement of health and care in these patients.

Among the bariatric surgery techniques, it is emphasized the vertical gastrectomy (VG) or gastric sleeve, which promotes a resection of the entire stomach fundus, allowing a reduction in gastric chamber, culminating in food restriction and hormonal decrease of ghrelin, providing acceleration of the gastrointestinal tract, allowing the continued use of HAART^14^. This procedure has proved to be a safety feature, standing out by the considerable reduction of weight in patients with HIV and obesity without causing complications or loss.

 The Federal Council of Medicine (CFM) in Brazil states that the indication and performance of bariatric surgery in adults should occur through the obesity diagnosis set about five years before, in situations where conventional treatments (diet, physical activity and pharmacotherapy) did not generate results, with such specialized treatment lasting at least two years. To undergo surgery, all patients must have a BMI greater than 40 kg/m² or greater than 35 kg/m² with associated comorbidities that bring harm to life, such as type 2 diabetes, obstructive sleep apnea, arterial hypertension, dyslipidemia, coronary heart disease, osteoarthritis, among others.

Thus, there is a deficit in the scientific environment, requiring studies to be conducted to assess which bariatric procedures are better for patients with morbid obesity and HIV. 

Based on the foregoing and on the difficulties, it was idealized to conduct this study in order to review the scientific literature on the applicability of VG in patients with HIV and morbid obesity.

## METHOD

### Defining the question

In this context of doubts, the following question was emerged: is the sleeve gastrectomy an effective surgical technique for patients with morbid obesity and HIV? 

### Seeking evidence

It was conduced a search in the electronic databases of national and international data of articles published from 1998 to 2015, those being: Scopus, US National Library of Medicine/National Institutes of Health (Pubmed), Cumulative Index to Nursing and Allied Health Literature (Cinahl), Scientific Electronic Library Online (Scielo), Cochrane and Lilacs. The descriptors of Medical Subject Heading (MeSH) used to collect the data were "Gastrectomy" and "Morbid obesity", combined by the operator "AND", with the descriptor "HIV", and their respective descriptors, in Portuguese and English. In this first search moment it was found a great amount of articles, being 2,148 in Scopus, 1234 Medline and 784 Cinahl.

### Revising and selecting the studies 

Shortly after the search, the inclusion criteria for the selection of articles were established as follows: full papers in Portuguese, English, available free of charge; theme related to the studied subject. Thus, studies that did not meet the relevant criteria were excluded, as dissertations, theses and editorials, among others.

### Assessment to articles quality 

**FIGURE 1:**
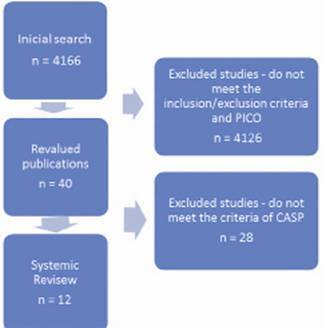
Route of systematic review showing the steps for articles selection

The review process was divided into two stages. First, it was used the Jadad Quality Scale consisting of five criteria with a five points total score. Articles lower than three points were considered with poor methodological quality and little possibility of extrapolating the results to clinical practice. Only 40 articles were included. Second, articles selected in the first stage were revalued using a form for studies evaluation, made by the Critical Appraisal Skills Programme (CASP). Studies that achieved a score of seven out of ten points were included in the sample, reaching the amount of 12 articles, as shown in ^Figure1^.

### Presenting the results

**FIGURE 2:**
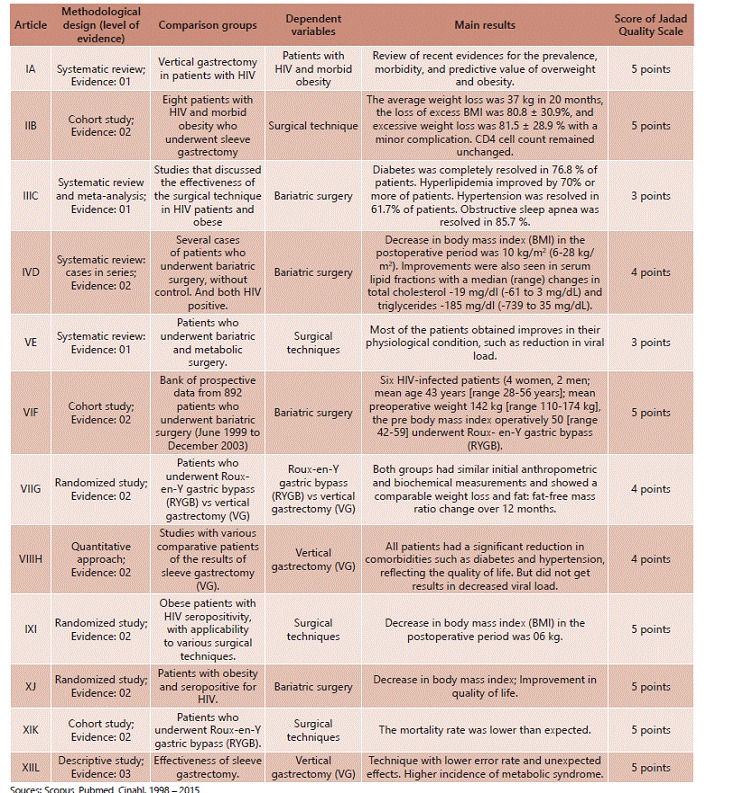
List of items that belong to the axis of the studied subject, according to the criteria

Finally, the articles included in the systematic review were categorized according to the following variables: methodological design (level of evidence), comparison groups, dependent variables and main results. Figure 2 shows all its variables.

## RESULTS

After analyzing all 12 articles, by not probabilistic calculation based on the frequency of surgical techniques, it was noticed that the most placed procedures were: 72.8% biliopancreatic derivation or duodenal switch, 71.2% vertical gastroplasty, 64, 8%, gastric bypass and 51.2% adjustable gastric banding.

Accordingly, it was observed that the vertical gastroplasty was among the most prevalent against other surgical techniques. Another point of relevance were the postoperative results, where it was noticed that most obese and HIV patients had a decrease in viral load, better control of type 2 diabetes and hypertension, and better immune response, as shown in Table 1.

**TABLE 1:** Distribution of positive results related to VG

Positive Results	Article	n	%
Immune system	1, 4, 5	3	10,3
Diabetes and hypertension	1,11	2	7,0
Diabetes, weight	1, 3, 6, 7,9,10	6	22,1
Viral charge	1, 7	2	6,9
CD4 level	1, 4	2	6,9
Diabetes and hypertension, viral load	6, 7	2	10,3
CD4 level, Diabetes	1	1	3,5
Hypertension	1	1	3,5
Immune system, diabetes and hypertension	1	1	3,5
Weight	4	1	6,9
Immune system, diabetes, weight	2	1	3,5
Hypertension, weight	5, 7, 8	3	10,3
Viral load, CD4 level	6, 8	2	6,9
Immune system, diabetes, weight, CD4 Level	6, 8	2	6,9
TOTAL		29	100,0

## DISCUSSION

Vertical gastrectomy, also called sleeve gastrectomy or longitudinal gastrectomy, was proposed as part of a biliopancreatic derivation without distal gastrectomy preserving the pylorus and reducing the ulcerogenic potential².

The aforementioned practice leads to a reduction of possible comorbidities associated with patients who have obesity and seropositivity for HIV, such as type 2 diabetes mellitus, hypertension, hypertrophic cardiomyopathy, hyperlipidemia, cholelithiasis, obstructive sleep apnea, hypoventilation, degenerative arthritis, psychosocial misfits, various types of neoplasms, chronic back pain, among others. Thus, it can be seen in studies IA, IIB and XN patients who went through surgical procedure with lower levels of viral load, thereby modifying the drug regimen with better physiological condition for therapeutical support^1^ ^,^ ^3^

VG acts as a gastric restriction (with removal of 70 to 80% of the proximal stomach) associated with a hormone component (ghrelin reduction) and accelerated intestinal transit^5^. Among the advantages of this procedure there is the lack of duodenum exclusion from intestinal transit, therefore not interfering with iron, calcium, zinc and B vitamins absorption sites, relevant for clinical compensation in AIDS patients. It can be converted to a procedure with malabsorptive component, such as gastric bypass Roux-en-Y and biliopancreatic diversion with duodenal switch in case of failure, also allowing access to bile and pancreatic ducts by usual endoscopic methods^8^.

Thus, the VG is one of those safe and effective methods, with low morbidity and mortality, good postoperative results and low complication rate. Furthermore, it can be used as the initial isolated or secondary treatment - for example, after gastric band fails. Thereby, this technique is being accepted and proposed by many as an isolated bariatric surgery, especially in patients with AIDS, for allowing the continued antiretroviral therapy^10^ ^,^ ^16^.

However, despite the possibility of maintaining the continuity of the intestinal transit, some studies have demonstrated the potential risk of postoperative long-term nutritional deficiencies such as decrease in the absorption of vitamin B12 and iron^19^. It also gives chance to gastroesophageal reflux "de novo"^9^, difficulty in gastric emptying and suture line fistula (2.7%), therefore needing multidisciplinary follow up to the success of surgery^4^.

Its necessary, however, to have in the future follow-up studies of complications in the late postoperative period to fully clarify the applicability of the surgical procedure in question. Nowadays, the experience in these patients with this procedure has proven quite effective, minimizing comorbidities that compromise biological and psychological development. 

## CONCLUSION

Vertical gastrectomy proved to be a safe procedure, favoring weight loss and control of morbid conditions associated with obesity and AIDS.
